# Immuno-Inflammatory Profiling and Complement Activation in Fabry Disease: A Cross-Sectional Study

**DOI:** 10.3390/ijms27156850

**Published:** 2026-07-30

**Authors:** Mercedes Peña-Rodríguez, Nuria Bara-Ledesma, Martin Fabregate, Montserrat Morales Conejo, Fernando Domínguez-Rodríguez, Borja Merino-Ortiz, Sinziana Stanescu, Pedro Ruiz-Sala, Andrés González García, Amaya Belanger-Quintana, Mónica López-Rodríguez

**Affiliations:** 1 Internal Medicine Department, Hospital Universitario Ramón y Cajal, IRYCIS, 28034 Madrid, Spain; 2 Faculty of Medicine and Health Sciences, Universidad de Alcalá (UAH), 28805 Alcalá de Henares, Spain; 3 Group of Multisystemic Diseases, IRYCIS, 28034 Madrid, Spain; 4 Unit for Congenital Metabolic Diseases and Other Rare Diseases, Internal Medicine Department, Reference Center for Inherited Metabolic Disease-MetabERN, Hospital Universitario 12 de Octubre, 28041 Madrid, Spain; 5 Centre for Biomedical Network Research on Rare Diseases (CIBERER), Instituto de Salud Carlos III, 28029 Madrid, Spain; 6 Cardiology Department, Hospital Universitario Puerta de Hierro, 28222 Majadahonda, Spain; 7 CSUR de Enfermedades Metabólicas, Hospital Universitario Ramón y Cajal, IRYCIS, 28034 Madrid, Spain; 8 Metabolic Diseases Unit, Department of Pediatrics, European Reference Network for Hereditary Metabolic Disorders (MetabERN), Hospital Universitario Ramón y Cajal, IRYCIS, 28034 Madrid, Spain; 9 Centro de Diagnóstico de Enfermedades Moleculares (CEDEM), Autonomous University of Madrid (UAM), IdiPaz, 28049 Madrid, Spain

**Keywords:** Fabry disease, low-grade systemic inflammation, fibrinogen, soluble TNF receptor 2, complement system, lysosomal storage disorders, LASSO, clustering analysis

## Abstract

Fabry disease (FD) is a rare X-linked lysosomal storage disorder caused by α-galactosidase A deficiency, leading to glycosphingolipid accumulation and progressive organ damage. Beyond substrate storage, low-grade inflammation and complement activation have been increasingly implicated in FD pathogenesis, yet a comprehensive characterization of this immuno-inflammatory profile is lacking. In this exploratory cross-sectional study, fifteen patients with FD and fifteen age- and sex-matched healthy controls (HCs) were assessed using a broad panel of systemic inflammatory, humoral immunity/complement, hematological, and endothelial biomarkers, integrated through univariate analysis (Cliff’s delta, δ), penalized least absolute shrinkage and selection operator (LASSO) regression, and unsupervised hierarchical clustering. Fibrinogen (δ = 0.60, 95% confidence interval (CI) 0.24–0.88), sTNFR2 (δ = 0.48, 95% CI 0.08–0.80), complement C4 (δ = 0.53, 95% CI 0.13–0.84), and lymphocyte count (δ = 0.52, 95% CI 0.15–0.85) showed the largest between-group effect sizes among the immuno-inflammatory biomarkers assessed, with higher levels in FD. Fibrinogen and sTNFR2 were the most stable predictors in LASSO bootstrap resampling (selected in 75.0% and 69.5% of iterations, respectively), and unsupervised clustering segregated FD from HCs with high accuracy (90% FD enrichment in the high-biomarker cluster; *p* < 0.001). These convergent findings indicate that FD is characterized by a distinct low-grade immuno-inflammatory profile dominated by innate immune and complement activation.

## 1. Introduction

Fabry disease (FD) is a rare X-linked lysosomal storage disease caused by partial or complete deficiency of α-galactosidase A activity, secondary to pathogenic variants in the GLA gene. This enzymatic defect leads to progressive intracellular accumulation of globotriaosylceramide (Gb3) and related glycosphingolipids in multiple cell types, including podocytes, neurons, and cardiomyocytes. This intracellular storage disrupts cellular homeostasis and signaling pathways, thereby contributing to multisystem organ dysfunction [1]. The estimated prevalence of FD is approximately 1:117,000 live births, though potentially higher when attenuated and later-onset forms are included, FD represents one of the most prevalent rare lysosomal storage disorders [2,3]. While the classic severe phenotype in hemizygous males has long been described, FD is now recognized as a clinically heterogeneous disorder spanning a broad spectrum of disease manifestations and severity in both males and females [4]. Major clinical manifestations include neuropathic pain, gastrointestinal disturbances, heat intolerance, ischemic stroke, and gradual kidney and heart involvement [1].

Lysosomal Gb3 accumulation has been proposed to act as a damage-associated molecular pattern (DAMP), thereby triggering downstream inflammatory cascades. At the mechanistic level, Gb3 deposition promotes oxidative stress, with reactive oxygen species (ROS) acting as upstream activators of the NF-κB pathway and its downstream cytokine production, establishing a Gb3 → ROS → NF-κB → cytokine axis that amplifies cellular injury [5,6]. Elevated circulating levels of proinflammatory cytokines have been reported in patients with FD [7,8], and increased TLR4-dependent cytokine expression in peripheral blood mononuclear cells further supports the involvement of innate immune pathways [9]. Evidence from clinical studies has additionally demonstrated complement system activation in FD patients, with elevated C3a and C5a levels persisting despite enzyme replacement therapy (ERT), reinforcing the role of innate immune dysregulation in disease pathogenesis [10,11]. Beyond FD, dysregulation of the TNF–TNFR signaling axis has been increasingly recognized as a shared inflammatory mechanism across lysosomal storage disorders, suggesting that substrate accumulation may converge on common immune effector pathways [12]. This sustained immune activation may persist independently of substrate storage, potentially driving progressive tissue injury despite ERT [13]. However, the overall immuno-inflammatory landscape in FD remains incompletely characterized, and the relative contribution of specific inflammatory pathways has yet to be fully elucidated.

Within this immuno-inflammatory framework, the vascular endothelium emerges as a central cellular compartment affected by Gb3 accumulation in FD. Accumulation of Gb3 within endothelial cells impairs nitric oxide bioavailability, upregulates adhesion molecules, and promotes a pro-thrombotic and pro-inflammatory phenotype, collectively establishing a state of chronic endothelial dysfunction that underlies the systemic vasculopathy characteristic of FD [14]. This endothelial injury is further amplified by the complement-mediated mechanisms described above, with elevated C3a and C5a promoting leukocyte recruitment and worsening vascular damage [11]. Despite this mechanistic complexity, the immuno-inflammatory characterization of FD has largely relied on isolated biomarker measurements, predominantly globotriaosylsphingosine (lyso-Gb3) and individual cytokines, and a comprehensive, simultaneous assessment of circulating inflammatory, complement, humoral, and endothelial markers in a single cohort remains lacking. Such an integrative approach is particularly warranted given the multisystem nature of FD and the recognized limitations of single-biomarker strategies in capturing the full extent of underlying immune dysregulation.

The aim of this exploratory cross-sectional study was to comprehensively characterize the circulating immuno-inflammatory profile of patients with FD compared with matched healthy controls (HCs). To this end, we employed a broad panel of systemic inflammatory, humoral immunity, complement, hematological, and endothelial biomarkers, integrated through a multi-level analytical approach designed to capture both individual biomarker differences and higher-order immunological patterns.

## 2. Results

### 2.1. Description of Patient Cohorts

Fifteen patients with FD were enrolled, comprising 4 males and 11 females, with a median age of 44 years [interquartile range (IQR), 38–50]. Patients sharing the same genetic variant were confirmed relatives. All male patients presented with a classical phenotype, whereas female patients showed more heterogeneous clinical characteristics. Renal involvement was documented in nine patients (60%), cardiac involvement in five (33%) and cerebrovascular involvement in three (20%), while six (40%) had no major organ involvement. Three patients (20%) presented concomitant involvement of all three territories. Seven patients (46%) were receiving FD-specific treatment, including ERT with agalsidase alfa or substrate reduction therapy (SRT), and none were receiving chaperone therapy. Detailed patient characteristics, including genetic features, are described in Table 1. Fifteen age- and sex-matched healthy controls were also included, with a median age of 45 years [IQR, 36–51]. Characteristics of subjects included in the HC cohort are described in Supplementary Table S1.

### 2.2. Univariate Biomarker Analysis

**Figure 1:**
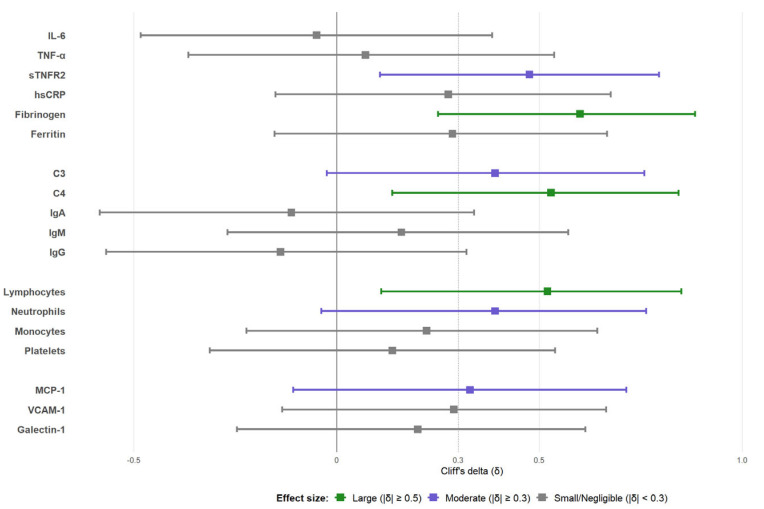
Forest plot of circulating immuno-inflammatory biomarkers comparing patients with Fabry disease and healthy controls. Square symbols represent the point estimates of the non-parametric effect size Cliff’s delta (δ), while horizontal bars indicate the corresponding 95% bootstrap confidence intervals. A δ value of 0 indicates no difference between groups; positive values denote higher biomarker concentrations in patients with FD, whereas negative values indicate higher concentrations in HCs. Biomarkers with |δ| values exceeding 0.3 and 0.5, corresponding to moderate and large effect sizes, respectively, are highlighted in green and purple. hsCRP: High-Sensitivity C-Reactive Protein; IgA: Immunoglobulin A; IgM: Immunoglobulin M; IgG: Immunoglobulin G; IL-6: Interleukin-6; MCP-1: Monocyte Chemoattractant Protein-1; TNF-α: Tumor necrosis factor α; sTNFR2: soluble tumor necrosis factor α receptor 2; VCAM-1: Vascular cell adhesion molecule-1.

**Figure 2:**
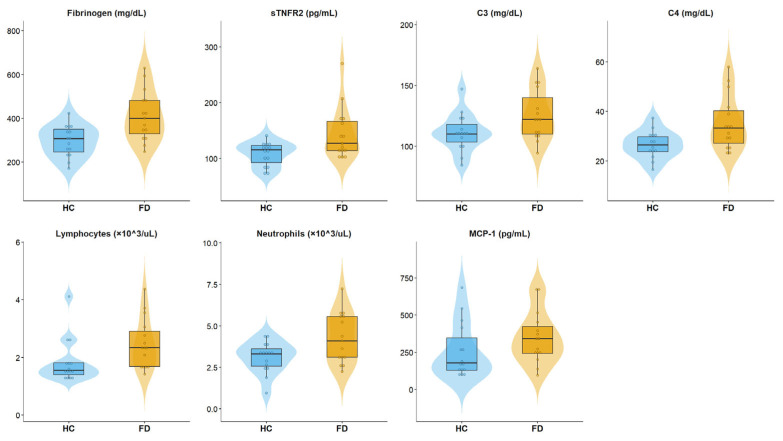
Distribution of plasma biomarker levels exhibiting moderate-to-large effect sizes (|δ| ≥ 0.3) in patients with Fabry disease (FD, orange) and healthy controls (HCs, blue). MCP-1: Monocyte Chemoattractant Protein-1; sTNFR2: soluble tumor necrosis factor α receptor 2.

Exploratory univariate analyses comparing circulating biomarkers between patients with FD and HCs are summarized in Table 2. The FD-specific biomarker plasma lyso-Gb3 showed the largest effect size across the entire panel (δ = 0.85, 95% confidence interval (CI): 0.53 to 1.00), consistent with the biochemical characterization of the cohort. Among markers of systemic inflammation, fibrinogen showed the largest between-group effect size (δ = 0.60, 95% CI: 0.24 to 0.88), followed by soluble TNF receptor 2 (sTNFR2, δ = 0.48, 95% CI: 0.08 to 0.80). Complement components C4 (δ = 0.53, 95% CI: 0.13 to 0.84) and C3 (δ = 0.39, 95% CI: 0.01 to 0.75) also displayed moderate-to-large between-group differences. Among hematological parameters, lymphocyte counts showed a large effect size (δ = 0.52, 95% CI: 0.15 to 0.85), whereas neutrophil counts showed a moderate effect size (δ = 0.39, 95% CI: −0.03 to 0.75). Circulating biomarkers related to endothelial dysfunction exhibited smaller between-group differences overall, with MCP-1 showing the largest effect size within this category (δ = 0.33, 95% CI: −0.12 to 0.72). The magnitude and direction of effect sizes for circulating immuno-inflammatory biomarkers across groups are depicted in Figure 1. Distributions of biomarkers exhibiting a moderate-to-large effect size are illustrated in Figure 2.

### 2.3. Penalized Multivariable Biomarker Selection

**Figure 3:**
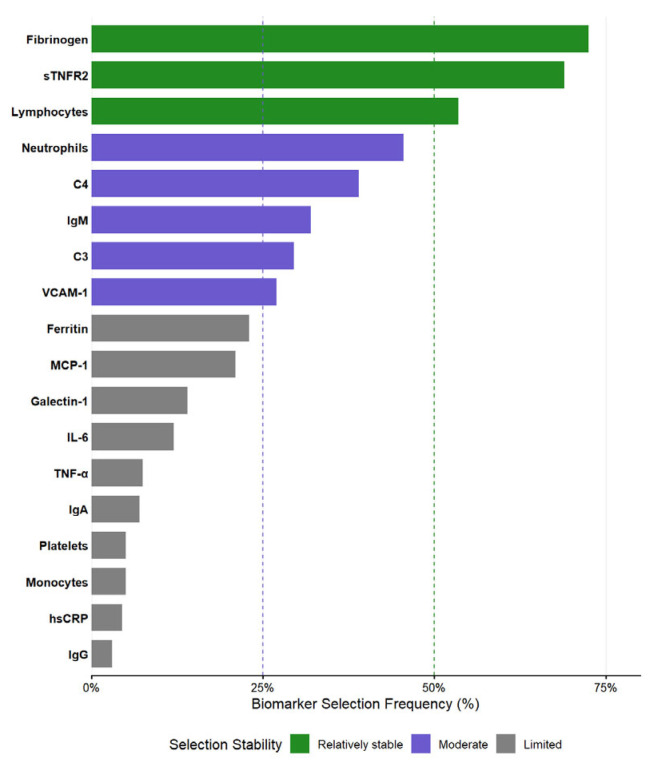
Stability of biomarker selection using LASSO logistic regression. Bar plot showing the frequency with which each biomarker was selected across 200 bootstrap resamples in the LASSO logistic regression model comparing patients with Fabry disease and healthy controls. Selection frequency represents the proportion of bootstrap iterations in which the biomarker was retained in the model using the penalty within one standard error of the minimum cross-validated deviance. Only biomarkers selected in at least one bootstrap iteration are displayed. Selection frequencies ≥50% were interpreted as relatively stable (green), and frequencies between 25% and 50% as moderate (purple). hsCRP: High-Sensitivity C-Reactive Protein; IgA: Immunoglobulin A; IgM: Immunoglobulin M; IgG: Immunoglobulin G; IL-6: Interleukin-6; MCP-1: Monocyte Chemoattractant Protein-1; TNF-α: Tumor necrosis factor α; sTNFR2: soluble tumor necrosis factor α receptor 2; VCAM-1: Vascular cell adhesion molecule-1.

A supervised penalized regression analysis identified a restricted subset of biomarkers concentrating most of the multivariable separation between FD and HCs (Figure 3). Fibrinogen and sTNFR2 showed the highest selection stability, being retained in 75% and 70% of bootstrap iterations, respectively. Lymphocyte (50%) and neutrophil (46.0%) counts also demonstrated moderate selection frequencies. Complement-related biomarkers, including C3 (29%) and C4 (28%), together with ferritin (28%) and IgM (26%), were selected less consistently but remained above the pre-specified stability threshold (25%).

### 2.4. Clustering Analysis of Circulating Biomarkers

**Figure 4:**
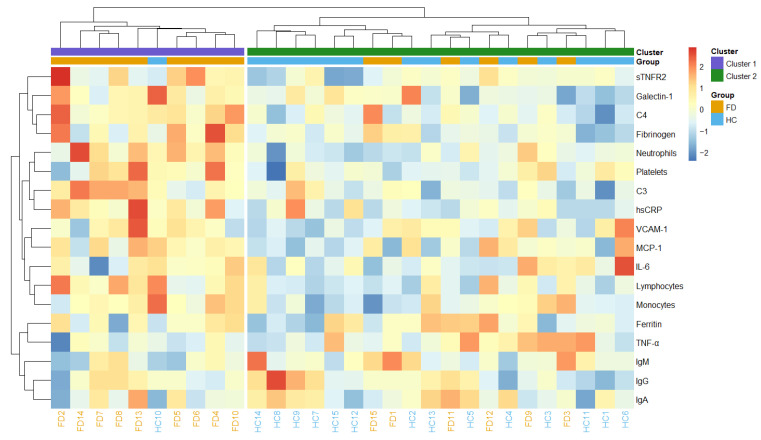
Heatmap and hierarchical clustering of circulating biomarker levels in patients with Fabry disease (FD) and healthy controls (HCs). The heatmap displays standardized biomarker levels (Z-scores), with rows corresponding to biomarkers and columns to individual subjects. Color intensity reflects relative biomarker levels, with red indicating higher and blue indicating lower standardized values. Unsupervised hierarchical clustering is shown by the column dendrogram, illustrating the segregation of subjects based on their biomarker profiles; the top annotation bars denote cluster (purple: cluster 1; green: cluster 2) and clinical group (orange: patients with FD; blue: HC). Row clustering groups biomarkers according to similarity in their expression patterns. hsCRP: High-Sensitivity C-Reactive Protein; IgA: Immunoglobulin A; IgM: Immunoglobulin M; IgG: Immunoglobulin G; IL-6: Interleukin-6; MCP-1: Monocyte Chemoattractant Protein-1; TNF-α: Tumor necrosis factor α; sTNFR2: soluble tumor necrosis factor α receptor 2; VCAM-1: Vascular cell adhesion molecule-1.

Unsupervised hierarchical clustering of the combined study population identified two distinct clusters, showing differential enrichment by clinical group (Figure 4). Cluster 1 (n = 10) was characterized by overall higher biomarker levels and was strongly enriched for patients with FD (9/10; 90%). In contrast, cluster 2 (n = 20) was predominantly composed of HCs (14/20; 70%). The association between cluster membership and clinical group was statistically significant (*p* < 0.001).

Neutrophil count (15.1%), C3 (11.4%), and C4 (10.9%) represented the main contributors to cluster separation, jointly explaining more than one-third of the total dissimilarity between clusters. Lymphocyte count (10.5%), fibrinogen (7.8%), sTNFR2 (7.5%), and platelet count (6.4%) also contributed substantially to cluster segregation. The relative contribution of individual biomarkers to inter-cluster dissimilarity are described in Supplementary Table S2. The demographic, genetic, and clinical characteristics of patients with FD according to cluster assignment are presented in Supplementary Table S3. Concomitant renal, cardiac, and cerebrovascular involvement was observed exclusively among patients assigned to cluster 1 (33% vs. 0%). To complement the cluster-level analysis, biomarker profiles were further examined within each age- and sex-matched FD–HC pair using a side-by-side heatmap (Figure S1).

## 3. Discussion

The present study identifies a distinct immuno-inflammatory profile in patients with FD, characterized by low-grade activation of innate immune and complement pathways rather than by marked elevations of classical proinflammatory cytokines. Across all three analytical approaches, fibrinogen, sTNFR2, complement C4, and lymphocyte count consistently emerged as the most robustly prioritized biomarkers, collectively defining the core immuno-inflammatory signature of the present cohort. Convergent signals were additionally observed for C3 and neutrophil count, further supporting a broader pattern of innate immune and complement engagement in FD.

Chronic inflammation has increasingly been recognized as a relevant contributor to the pathophysiology of FD, complementing the classical paradigm centered on lysosomal glycosphingolipid accumulation. Experimental and clinical studies have consistently reported evidence of immune activation in FD, including increased levels of circulating inflammatory mediators, activation of innate immune pathways, and oxidative stress, even in patients receiving disease-specific therapies [15,16]. Cytokines are key mediators of innate immunity, and elevated plasma levels of proinflammatory cytokines in FD have been documented in multiple clinical and in vitro studies [8,9]. Elevations of IL-6 and TNF-α in FD are frequently reported, but the magnitude of these elevations and their relationship with treatment vary across cohorts [17]. A notable finding in the present cohort was the significant elevation of sTNFR2 in the absence of marked increases in classical proinflammatory cytokines, including IL-6 and TNF-α. This apparent discrepancy may be explained by several factors, including differences in cohort composition, disease severity, and treatment exposure, as well as methodological aspects related to cytokine quantification. In particular, cytokines such as IL-6 and TNF-α have short half-lives and marked biological variability, which may limit their utility as stable biomarkers of chronic inflammation. Our findings are consistent with this interpretation, as sTNFR2 is a more integrative and temporally stable marker of TNF pathway activation [18]. Furthermore, experimental evidence suggests that sTNFR2 can bind circulating TNF-α and stabilize it, thereby prolonging its half-life and potentially enhancing its biological activity [19].

This interpretation is further supported by extensive evidence from other chronic inflammatory conditions in which soluble TNF receptors have emerged as robust biomarkers of disease activity and prognosis. In diabetic nephropathy and chronic kidney disease, elevated circulating levels of sTNFR1 and sTNFR2 strongly predict accelerated decline in glomerular filtration rate, progressive CKD, and cardiovascular events, independently of traditional risk factors and glycemic control [20]. Similarly, in spondyloarthritis and other chronic inflammatory settings, increased sTNFR2 levels have been associated with more severe disease and with an increased turnover of the TNF–TNFR2 axis, reflecting sustained pathway activation rather than transient cytokine release [21]. Collectively, these observations support the concept that sTNFRs act as integrative markers of chronic TNF pathway engagement, supporting their relevance for capturing persistent, low-grade inflammation in FD, even when circulating TNF-α levels appear unremarkable.

In line with this low-grade inflammatory profile, other circulating biomarkers of systemic inflammation further supported the presence of chronic immune activation in FD. In the present cohort, fibrinogen levels were significantly higher in patients with FD than in HCs, with moderate-to-large effect size, indicating a sustained inflammatory state. Notably, fibrinogen was also the most consistently selected biomarker across multivariable analyses. Similar findings have been reported previously, with fibrinogen elevations observed in patients with FD, particularly in those with cardiovascular or renal involvement, and were interpreted as markers of chronic vascular inflammation rather than acute inflammatory responses [22]. In contrast, hsCRP showed non-significant differences in our study, a result that is congruent with several published reports describing normal or minimally increased CRP levels in FD cohorts [13]. This variability across studies suggests that CRP may lack sufficient sensitivity to capture the subtle and persistent inflammatory burden characteristic of FD.

Beyond soluble mediators, innate immune effector systems appear to amplify and perpetuate inflammatory signals triggered by glycosphingolipid accumulation. Among these, the complement system constitutes a key interface between metabolic disturbance and immune activation, bridging chronic inflammatory stimuli with vascular dysfunction, leukocyte recruitment, and tissue injury. Previous studies have already described significantly elevated levels of C3a, C3, inactivated C3b (iC3b), C5a, C4, complement 1q subcomponent c (C1qc) and complement factor B in humans and animals with FD compared with HCs [10,11,23]. In our study, since immunoglobulin levels (IgG, IgA, and IgM) did not differ, the elevation of C4 points to activation mediated by DAMPs, such as Gb3, rather than by immune complex deposition. The relevance of this finding is underscored by its consistency across all analytical levels. C4 factor was not only significantly elevated in the univariate analysis but also emerged as a high-importance variable in the multivariable analysis and played a decisive role in the unsupervised clustering of patients with FD. While C3, the central convergence point of the cascade, showed a similar upward trend, it did not reach statistical significance, likely due to our limited sample size. Collectively, these findings suggest a shift in the FD paradigm, emphasizing the predominance of innate effector systems over adaptive humoral mechanisms in disease pathogenesis.

An additional and noteworthy finding of the present study was the significant increase in circulating lymphocyte counts in patients with FD compared with healthy controls, with a moderate effect size. This observation is in line with previous reports describing increased percentages of total lymphocytes and B cells in FD patients [24], and is biologically plausible within the context of chronic, low-grade immune activation and complement engagement documented in this disorder. Mechanistically, the complement-derived anaphylatoxins C3a and C5a are known to exert direct immunomodulatory effects on adaptive immune cells: C5a and C3a interact with their respective G protein-coupled receptors on both antigen-presenting cells and T cells, providing costimulatory and survival signals that promote lymphocyte proliferation and differentiation [25]. Although the specific lymphocyte subpopulations responsible for this expansion were not characterized in the present study, the pattern of complement activation and innate immune engagement observed here raises the hypothesis that regulatory T cells, NK cells, or B cells, all known targets of complement anaphylatoxin signaling, may contribute to the observed lymphocytosis. Complement dysregulation also promotes the overexpression of MCP-1 and VCAM-1, contributing to organ damage. However, VCAM-1 or MCP-1 levels did not differ significantly in the present cohort, negative findings possibly reflecting its clinical heterogeneity and the predominance of female patients with milder phenotypes. Consistent with our findings, transcriptomic studies have identified dysregulation of immune-related pathways in FD, supporting the relevance of immuno-inflammatory processes in disease pathophysiology [26,27].

We also explored whether the immuno-inflammatory profile identified by hierarchical clustering was associated with specific clinical features. Patients assigned to the FD-enriched cluster showed more extensive organ involvement and were the only individuals with concomitant renal, cardiac, and cerebrovascular disease, whereas this pattern was not observed in the HC-predominant cluster. These findings are biologically plausible and suggest that this biomarker profile may be more closely associated with the cumulative burden of disease than with the conventional classification into classical and later-onset phenotypes. The partial overlap between clinical groups and clusters further illustrates the biological heterogeneity of FD. This variability may reflect differences in disease severity, sex-related heterogeneity associated with X-chromosome inactivation, or the effects of disease-specific therapy [1]. Conversely, the presence of one HC within the FD-enriched cluster indicates that, although the identified biomarker signature discriminates between groups at the population level, it should not be interpreted as a diagnostic signature for individual patients.

This study has several limitations that should be acknowledged. The relatively small sample size, the predominance of milder phenotypes, and the overrepresentation of female patients may have reduced statistical power to detect subtle differences. However, this heterogeneity may also enhance the clinical representativeness of the study population by capturing a broader spectrum of disease phenotypes. Importantly, biomarkers with higher selection frequencies largely overlapped with those showing moderate to large univariate effect sizes and coherent biological directionality, including markers of systemic inflammation, innate immune activation, and hematological alterations. This convergence across univariate, unsupervised, and penalized multivariable analyses supports the robustness of these biomarkers as key contributors to the immuno-inflammatory profile associated with FD. These results should be interpreted as prioritization signals rather than definitive multivariable models, given the exploratory nature of the analysis and the limited sample size. Finally, the study was restricted to a predefined panel of circulating biomarkers, providing only a partial view of the biological complexity of FD. Future studies integrating targeted biomarker analyses with complementary high-throughput approaches may provide a more comprehensive characterization of disease mechanisms.

## 4. Materials and Methods

### 4.1. Study Design and Population

This was an exploratory, cross-sectional, single-center observational study. Consecutive adult patients with genetically or enzymatically confirmed FD were prospectively enrolled at Hospital Universitario Ramón y Cajal (Madrid, Spain) between September 2022 and April 2023. HCs were recruited concurrently with FD patients from voluntary blood donors and non-affected relatives or accompanying persons attending the hospital, with no personal history of FD or other relevant chronic conditions, including renal, cardiovascular or cerebrovascular disease. HCs were individually matched 1:1 to patients with FD by age (±5 years) and sex. Exclusion criteria for both groups included autoimmune or autoinflammatory diseases, solid organ transplantation, major cardiovascular events or surgery within 90 days before inclusion, severe comorbidities, or any condition potentially interfering with study objectives.

The study (NCT06007768) was approved by the institutional Ethics Committee (reference 2021/284) and conducted in accordance with the Declaration of Helsinki and applicable national regulations. All participants provided written informed consent prior to inclusion.

### 4.2. Data Collection and Definitions

Demographic characteristics (sex and age) were collected for all participants. For patients with FD, clinical data including genetic variant analysis, clinical phenotype, and FD-specific treatment were also recorded. FD patients were classified as having a Classic or Later-Onset phenotype [28]. Major organ involvement was defined as the presence of renal, cardiac, and/or cerebrovascular disease attributable to FD. Renal involvement was defined as the presence of proteinuria or a decreased glomerular filtration rate (eGFR < 60 mL/min) estimated with the CKD-EPI equation. Cardiac involvement was defined as the presence of left ventricular hypertrophy or cardiac arrhythmia. Cerebrovascular involvement was defined as ischemic lesions on magnetic resonance imaging, a history of stroke, or transient ischemic attack.

Biochemical and hematological analyses were performed at the certified institutional laboratory according to standard procedures, and included serum creatinine, complement components C3 and C4, IgA, IgG and IgM immunoglobulins, fibrinogen, high sensitive C-reactive protein, blood count and erythrocyte sedimentation rate. Spot urine samples were collected for urinary protein-to-creatinine ratio determination. Plasma lyso-Gb3 levels were quantified using liquid chromatography coupled to tandem mass spectrometry (LC–MS/MS) operating in multiple reaction monitoring mode. Plasma lyso-Gb3, rather than Gb3, was selected as the disease-specific biomarker in this study, given its higher analytical reliability and better correlation with cardiac disease measures across FD subpopulations [29].

Additional peripheral blood samples were collected in EDTA tubes, processed according to standard operating procedures, and stored at the BioBank Hospital Ramón y Cajal-IRYCIS (National Registry of Biobanks B.0000678), integrated in the Biobanks and Biomodels Platform of the ISCIII (PT20/00045). Subsequently, plasma aliquots were used for the determination of circulating biomarkers that could not be measured in the central laboratory. Monocyte chemoattractant protein-1 (MCP-1; ELISA kit, Cat. No. E-EL-H6005), galectin-1 (Cat. No.E-EL-H1051), vascular cell adhesion molecule-1 (VCAM-1; Cat. No. E-EL-H5587), interleukin-6 (IL-6; Cat. No. E-EL-H6156), tumor necrosis factor-α (TNF-α; Cat. No. E-EL-H0109), and soluble tumor necrosis factor receptor (sTNFR2; Cat. No. E-EL-H2436) were quantified using commercially available enzyme-linked immunosorbent assay (ELISA) kits (Elabscience Biotechnology Co., Ltd., Wuhan, Hubei, China), according to the manufacturer’s instructions.

### 4.3. Statistical Analysis

All statistical analyses were exploratory in nature. The sample size was determined by the number of eligible patients with genetically or enzymatically confirmed FD under active follow-up at our referral center during the recruitment period. No formal a priori sample size calculation was performed. Analyses were conducted using R Statistical Software (v4.5.2; R Core Team, Vienna, Austria), primarily with the *mice*, *glmnet*, *cluster*, and base packages.

A univariate analysis was performed to characterize differences in circulating biomarkers between patients with FD and HCs. Continuous variables were summarized as median and IQR, and categorical variables as absolute counts and percentages. Between-group comparisons were performed using the Mann–Whitney U test for continuous variables or Fisher’s exact test for categorical variables. Effect sizes were estimated using Cliff’s delta (δ), a non-parametric metric reflecting the probability that a randomly selected FD patient has a higher biomarker value than a HC minus the reverse probability. Effect sizes ranged from −1 to +1 and were interpreted as negligible/small (<0.3), moderate (0.3–0.5), or large (≥0.5) [30]. Percentile-based 95% confidence intervals (CIs) for δ were obtained by non-parametric bootstrap resampling (2000 iterations). Given the limited sample size, interpretation was guided primarily by effect sizes and their CIs. *p*-values are reported for descriptive purposes only, both raw and after false discovery rate adjustment (Benjamini–Hochberg).

A multivariable exploratory strategy combining complementary supervised and unsupervised approaches was applied to assess the convergence between biomarker-driven discrimination and intrinsic data structure. Penalized logistic regression using the least absolute shrinkage and selection operator (LASSO) was first applied to identify a reduced subset of biomarkers associated with group separation between FD and HCs. The regularization parameter was selected using the one-standard-error rule based on cross-validated deviance. Selection stability was evaluated by bootstrap resampling (200 iterations), with selection frequencies recorded for each candidate biomarker. For descriptive interpretation, frequencies ≥ 50% were considered relatively stable, and those between 25% and 50% moderately stable. In parallel, hierarchical clustering was performed as an unsupervised approach to identify intrinsic biomarker patterns independent of clinical group labels. Pairwise dissimilarities were computed using Euclidean distance, and clustering was conducted using Ward’s D2 linkage method. Cluster structure was further explored by quantifying the contribution of each biomarker to inter-cluster separation using standardized between-cluster differences.

Prior to both multivariate approaches, biomarkers underwent three sequential preprocessing steps: log-transformation, imputation of missing data, and standardization. First, biomarkers with non-normal distributions were log-transformed where appropriate. Then, variables with less than or equal to 20% missing data were handled using multiple imputation by chained equations (MICE) with predictive mean matching, under a missing-at-random assumption. A stochastic single imputation dataset was generated for downstream non-inferential analyses. Finally, biomarkers were Z-score standardized. Plasma lyso-Gb3 was used exclusively for descriptive characterization of the FD cohort and was not included in the multivariable analyses because of its established diagnostic performance.

## 5. Conclusions

The present study identifies a distinct, low-grade immuno-inflammatory profile in patients with FD particularly involving complement factors and specific proinflammatory mediators. The convergence of the univariate, supervised, and unsupervised analyses supports the robustness of this exploratory biomarker signature despite the relatively small and clinically heterogeneous cohort. Within the limitations of this study, the identified immuno-inflammatory signature appeared to be more closely associated with the cumulative burden of organ involvement than with the conventional classification into classical and later-onset phenotypes, suggesting that inflammatory activation may reflect disease severity rather than phenotype alone. Collectively, these findings support the concept that chronic innate immune activation may contribute to FD pathophysiology beyond lysosomal glycosphingolipid accumulation and identify complement components and sTNFR2 as candidate biomarkers for immuno-inflammatory characterization and potential adjunctive therapeutic targeting. Further studies in larger cohorts are needed to confirm these hypothesis-generating findings and to better define the role of inflammation in the pathogenesis and clinical progression of FD.

## Figures and Tables

**Table 1:** Demographic, genetic and clinical features by patient for the FD cohort.

Patient ID	Age (Years)	Sex	cDNA Aa Change Variant Identifier rsID	Variant Type	FD Phenotype	Major Organ Involvement	Lyso-Gb3 (nM)	FD Treatment
FD1	44	F	c.646dupT p.Y216fs rs1928199153	FS	Classical	Renal	7.97	SRT
FD2	68	F	c.646dupT p.Y216fs rs1928199153	FS	Classical	Cardiac Renal Cerebrovascular	5.65	NT
FD3	43	F	c.1088G>A p.R363H rs111422676	MS	Later-Onset	No	0.86	NT
FD4	44	M	c.646dupT p.Y216fs rs1928199153	FS	Classical	Cardiac Renal Cerebrovascular	42.37	ERT + SRT
FD5	48	M	c.646dupT p.Y216fs rs1928199153	FS	Classical	Cardiac Renal	83.63	ERT
FD6	38	F	c.646dupT p.Y216fs rs1928199153	FS	Classical	No	6.87	SRT
FD7	50	F	c.713G>A p.S238N rs730880450	MS	Later-Onset	Renal	2.11	NT
FD8	42	F	c.194G>A p.S65T rs104894848	MS	Later-Onset	Renal	10.05	NT
FD9	22	F	c.1088G>A p.R363H rs111422676	MS	Later-Onset	No	0.64	NT
FD10	65	F	c.658C>T p.R220X rs727503949	NS	Classical	Cardiac Renal Cerebrovascular	8.41	ERT
FD11	32	M	c.658C>T p.R220X rs727503949	NS	Classical	Cardiac Renal	7.88	ERT
FD12	65	F	c.1088G>A p.R363H rs111422676	MS	Later-Onset	No	0.80	NT
FD13	26	M	c.85dupG p.A29fs rs1569306181	FS	Classical	No	113.07	ERT
FD14	45	F	c.427G>A p.A143T rs104894845	MS	Later-Onset	No	0.39	NT
FD15	42	F	c.646dupT p.Y216fs rs1928199153	FS	Classical	Renal	13.29	NT

Aa change: amino acid change generated by the variant in the protein. cDNA: Complementary DNA variant according to Human Genome Variation Society nomenclature. ERT: enzyme replacement therapy. F: female. FS: Frameshift. Lyso-Gb3: globotriaosylsphingosine. M: male. MS: Missense. NS: Nonsense. NT: not treated. SRT: substrate reduction therapy.

**Table 2:** Differences in circulating biomarkers between patients with Fabry disease and healthy controls.

	**FD (*n* = 15)** **Median [IQR]**	**HCs (*n* = 15)** **Median [IQR]**	**Effect Size (δ)** **(95% CI)**	***p***	***p*** **-adj**
**FD-specific biomarker**					
Lyso-Gb3, nM	7.97 [0.86–13.29]	0.58 [0.55–0.61]	0.85 (0.53 to 1.00)	<0.001	0.002
**Systemic inflammation biomarkers**					
IL-6, pg/mL	0.89 [0.62–1.07]	0.91 [0.54–1.13]	−0.05 (−0.46 to 0.39)	0.838	0.838
TNF-α, pg/mL ^a^	12.5 [4.2–21.3]	6.4 [2.9–25.8]	0.07 (−0.38 to 0.53)	0.760	0.800
sTNFR2, pg/mL	127 [114–167]	116 [92–123]	0.48 (0.08 to 0.80)	0.026	0.132
hsCRP, ng/mL	1.1 [0.8–2.4]	0.7 [0.5–1.2]	0.28 (−0.14 to 0.67)	0.203	0.368
Fibrinogen, mg/dL	400 [328–481]	307 [245–351]	0.60 (0.25 to 0.89)	0.004	0.085
Ferritin, ng/mL ^b^	64.8 [28.4–117.5]	31.5 [13.6–70.4]	0.29 (−0.17 to 0.68)	0.201	0.368
**Humoral immunity/complement biomarkers**					
Complement C3, mg/dL	122 [110–140]	110 [104–118]	0.39 (−0.01 to 0.75)	0.071	0.203
Complement C4, mg/dL ^c^	33.3 [27.2–40.3]	26.5 [23.7–29.7]	0.53 (0.14 to 0.83)	0.016	0.109
IgA, UI/mL	189 [165–234]	242 [170–273]	−0.11 (−0.54 to 0.35)	0.619	0.687
IgM, UI/mL	117 [100–155]	103 [84–120]	0.16 (−0.24 to 0.58)	0.468	0.668
IgG, UI/mL ^d^	1100 [963–1100]	1100 [920–1275]	−0.14 (−0.59 to 0.29)	0.545	0.681
**Hematological parameters**					
Lymphocyte count, 10^3^/µL	2.34 [1.69–2.92]	1.56 [1.40–1.81]	0.52 (0.14 to 0.85)	0.015	0.109
Neutrophil count, 10^3^/µL	4.09 [3.11–5.57]	3.31 [2.58–3.62]	0.39 (−0.02 to 0.74)	0.071	0.203
Monocyte count, 10^3^/µL	0.48 [0.39–0.53]	0.37 [0.34–0.52]	0.22 (−0.23 to 0.61)	0.309	0.515
Platelet count, 10^3^/µL	277 [249–329]	267 [248–301]	0.14 (−0.33 to 0.56)	0.539	0.681
**Endothelial biomarkers**					
MCP-1, pg/mL	342 [243–424]	179 [129–346]	0.33 (−0.09 to 0.73)	0.130	0.325
VCAM-1, ng/mL	38.6 [30.0–42.9]	28.6 [23.5–39.8]	0.29 (−0.15 to 0.68)	0.187	0.368
Galectin-1, ng/mL	2.06 [1.71–2.53]	1.69 [1.19–2.56]	0.20 (−0.25 to 0.66)	0.361	0.556

^a^ Missing data for 1 HC. ^b^ Missing data for 1 FD patient. ^c^ Missing data for 1 HC. ^d^ Missing data for 1 FD and 1 HC. Between-group comparisons calculated using the two-sided Mann–Whitney U test, and *p*-values reported for descriptive purposes as raw (*p*) and after false discovery rate adjustment (*p*-adj). CI: Confidence interval; FD: Fabry disease; HCs: Healthy controls; hsCRP: High-Sensitivity C-Reactive Protein; IgA: Immunoglobulin A; IgM: Immunoglobulin M; IgG: Immunoglobulin G; IL-6: Interleukin-6; Lyso-Gb3: globotriaosylsphingosine; MCP-1: Monocyte Chemoattractant Protein-1; TNF-α: Tumor necrosis factor α; sTNFR2: soluble tumor necrosis factor α receptor 2; VCAM-1: Vascular cell adhesion molecule-1.

## Data Availability

The data presented in this study are available on request from the corresponding author. The data are not publicly available due to participant confidentiality.

## Supplementary Materials

The following supporting information can be downloaded at: https://www.mdpi.com/article/10.3390/ijms27156850/s1.

## References

[1] Germain D.P. (2010). Fabry disease. Orphanet J. Rare Dis..

[2] Meikle P.J., Hopwood J.J., Clague A.E., Carey W.F. (1999). Prevalence of Lysosomal Storage Disorders. JAMA.

[3] Desnick R.J., Ioannou Y.A., Eng C.M., Valle D.L., Antonarakis S., Ballabio A., Beaudet A.L., Mitchell G.A. (2001). α-Galactosidase A Deficiency: Fabry Disease. The Metabolic and Molecular Bases of Inherited Disease.

[4] Arends M., Wanner C., Hughes D., Mehta A., Oder D., Watkinson O.T., Elliott P.M., Linthorst G.E., Wijburg F.A., Biegstraaten M. (2017). Characterization of Classical and Nonclassical Fabry Disease: A Multicenter Study. J. Am. Soc. Nephrol..

[5] Rydzek J., Muzyka A., Majcherczyk K., Soczyńska J., Gawełczyk W., Żołyniak M., Woźniak S. (2026). Fabry Disease: A Focus on the Role of Oxidative Stress. Antioxidants.

[6] Simonetta I., Baglio I., Tuttolomondo A. (2026). Molecular, Metabolic and Inflammatory Patterns Involved in Pathogenesis of Anderson-Fabry Disease. Cells.

[7] Yogasundaram H., Nikhanj A., Putko B.N., Boutin M., Jain-Ghai S., Khan A., Auray-Blais C., West M.L., Oudit G.Y. (2018). Elevated Inflammatory Plasma Biomarkers in Patients With Fabry Disease: A Critical Link to Heart Failure With Preserved Ejection Fraction. J. Am. Heart Assoc..

[8] Yuan Y., Zhao Y., Li F., Ling C., Wu Y., Ma W., Wang Z., Yuan Y., Hao H., Zhang W. (2024). Inflammatory Cytokine Expression in Fabry Disease: Impact of Disease Phenotype and Alterations under Enzyme Replacement Therapy. Front. Immunol..

[9] De Francesco P.N., Mucci J.M., Ceci R., Fossati C.A., Rozenfeld P.A. (2013). Fabry Disease Peripheral Blood Immune Cells Release Inflammatory Cytokines: Role of Globotriaosylceramide. Mol. Genet. Metab..

[10] Laffer B., Lenders M., Ehlers-Jeske E., Heidenreich K., Brand E., Köhl J. (2024). Complement Activation and Cellular Inflammation in Fabry Disease Patients despite Enzyme Replacement Therapy. Front. Immunol..

[11] Magnusen A.F., Pandey M.K. (2024). Complement System and Adhesion Molecule Skirmishes in Fabry Disease: Insights into Pathogenesis and Disease Mechanisms. Int. J. Mol. Sci..

[12] Pandey M.K. (2023). Exploring Pro-Inflammatory Immunological Mediators: Unraveling the Mechanisms of Neuroinflammation in Lysosomal Storage Diseases. Biomedicines.

[13] Rozenfeld P., Feriozzi S. (2017). Contribution of Inflammatory Pathways to Fabry Disease Pathogenesis. Mol. Genet. Metab..

[14] Faro D.C., Di Pino F.L., Monte I.P. (2024). Inflammation, Oxidative Stress, and Endothelial Dysfunction in the Pathogenesis of Vascular Damage: Unraveling Novel Cardiovascular Risk Factors in Fabry Disease. Int. J. Mol. Sci..

[15] Biddeci G., Spinelli G., Colomba P., Duro G., Giacalone I., Di Blasi F. (2025). Fabry Disease Beyond Storage: The Role of Inflammation in Disease Progression. Int. J. Mol. Sci..

[16] Feriozzi S., Rozenfeld P. (2021). Pathology and Pathogenic Pathways in Fabry Nephropathy. Clin. Exp. Nephrol..

[17] Chen K.H., Chien Y., Wang K.L., Leu H.B., Hsiao C.Y., Lai Y.H., Wang C.Y., Chang Y.L., Lin S.J., Niu D.M. (2016). Evaluation of Proinflammatory Prognostic Biomarkers for Fabry Cardiomyopathy With Enzyme Replacement Therapy. Can. J. Cardiol..

[18] Paccalet A., Crola Da Silva C., Mechtouff L., Amaz C., Varillon Y., de Bourguignon C., Cartier R., Prieur C., Tomasevic D., Genot N. (2021). Serum Soluble Tumor Necrosis Factor Receptors 1 and 2 Are Early Prognosis Markers After ST-Segment Elevation Myocardial Infarction. Front. Pharmacol..

[19] Iversen P.L., Kipshidze N., Kipshidze N., Dangas G., Ramacciotti E., Kakabadze Z., Fareed J. (2023). A Novel Therapeutic Vaccine Targeting the Soluble TNFα Receptor II to Limit the Progression of Cardiovascular Disease: AtheroVaxTM. Front. Cardiovasc. Med..

[20] Moroney E., Niewczas M.A., Ekinci E.I., MacIsaac R.J. (2026). Soluble Tumor Necrosis Factor Receptors (STNFRs) as Biomarkers for Diabetic Kidney Disease. J. Diabetes Complicat..

[21] Venken K., Jarlborg M., Decruy T., Mortier C., Vlieghe C., Gilis E., De Craemer A.-S., Coudenys J., Cambré I., Fleury D. (2023). Distinct Immune Modulatory Roles of Regulatory T Cells in Gut versus Joint Inflammation in TNF-Driven Spondyloarthritis. Ann. Rheum. Dis..

[22] Schei J., Stefansson V.T.N., Mathisen U.D., Eriksen B.O., Solbu M.D., Jenssen T.G., Melsom T. (2016). Residual Associations of Inflammatory Markers with EGFR after Accounting for Measured GFR in a Community-Based Cohort without CKD. Clin. J. Am. Soc. Nephrol..

[23] Heo S.H., Kang E., Kim Y.M., Go H., Kim K.Y., Jung J.Y., Kang M., Kim G.H., Kim J.M., Choi I.H. (2017). Fabry Disease: Characterisation of the Plasma Proteome Pre- and Post-Enzyme Replacement Therapy. J. Med. Genet..

[24] Kurdi H., Lavalle L., Moon J.C.C., Hughes D. (2024). Inflammation in Fabry Disease: Stages, Molecular Pathways, and Therapeutic Implications. Front. Cardiovasc. Med..

[25] Strainic M.G., Liu J., Huang D., An F., Lalli P.N., Muqim N., Shapiro V.S., Dubyak G.R., Heeger P.S., Medof M.E. (2008). Locally Produced Complement Fragments C5a and C3a Provide Both Costimulatory and Survival Signals to Naive CD4^+^ T Cells. Immunity.

[26] Breznik N., Levstek T., Vujkovac B., Cokan Vujkovac A., Trebušak Podkrajšek K. (2025). Transcriptomic Approach in Understanding Fabry Nephropathy: A Review of the Literature and Proof-of-Concept. Genes.

[27] Coelho-Ribeiro B., Silva H.G., Sampaio-Marques B., Fraga A.G., Azevedo O., Pedrosa J., Ludovico P. (2024). Inflammation and Exosomes in Fabry Disease Pathogenesis. Cells.

[28] Nowak A., Mechtler T.P., Hornemann T., Gawinecka J., Theswet E., Hilz M.J., Kasper D.C. (2018). Genotype, phenotype and disease severity reflected by serum LysoGb3 levels in patients with Fabry disease. Mol. Genet. Metab..

[29] Ramaswami U., West M.L., Tylee K., Castillon G., Braun A., Ren M., Doobaree I.U., Howitt H., Nowak A. (2025). The use and performance of lyso-Gb3 for the diagnosis and monitoring of Fabry disease: A systematic literature review. Mol. Genet. Metab..

[30] Meissel K., Yao E.S. (2024). Using Cliff’s Delta as a Non-Parametric Effect Size Measure: An Accessible Web App and R Tutorial. Pract. Assess. Res. Eval..

