# Supplementary material for: Immuno-Inflammatory Profiling and Complement Activation in Fabry Disease: A Cross-Sectional Study
Source: Int J Mol Sci. 2026 Jul 30;27(15):6850. doi: 10.3390/ijms27156850 (PMC13466531; doi:10.3390/ijms27156850)
Supplement: Supplementary file 1 [file ijms-27-06850-s001.zip › ijms-4417831-supplementary.pdf]

Supplementary Information

Immuno-inflammatory Profiling and Complement Activation in Fabry Disease: A Cross-sectional Study

Supplementary Table S1. Characteristics by patient for the HCs cohort.

ID	Age	Sex	Lyso-Gb3, nM
HC1	47	F	0.59
HC2	67	F	0.57
HC3	45	F	0.61
HC4	44	M	0.55
HC5	49	M	0.59
HC6	36	F	0.56
HC7	51	F	0.55
HC8	44	F	0.66
HC9	24	F	0.58
HC10	69	F	0.56
HC11	33	M	0.58
HC12	68	F	0.61
HC13	28	M	0.67
HC14	47	F	0.49
HC15	39	F	0.54

HC: Healthy control. M: male; F: female.

**Supplementary Table S2.** Relative and cumulative contribution of circulating biomarkers to inter-cluster separation in the unsupervised hierarchical clustering analysis.			
	**Circulating biomarker**	**Contribution (%)**	**Cumulative contribution (%)**
**1**	Neutrophils	15.1%	15.1%
**2**	C3	11.4%	26.5%
**3**	C4	10.9%	37.5%
**4**	Lymphocytes	10.5%	48.0%
**5**	Fibrinogen	7.8%	55.8%
**6**	sTNF-R	7.5%	63.3%
**7**	Platelets	6.4%	69.7%
**8**	hsCRP	5.9%	75.6%
**9**	Galectin-1	5.5%	81.1%
**10**	Monocytes	5.4%	85.5%
**11**	MCP-1	3.7%	90.3%
**12**	TNF-α	3.6%	93.9%
**13**	IgM	2.6%	96.5%
**14**	VCAM-1	2.0%	98.5%
**15**	IgA	0.9%	99.4%
**16**	IgG	0.3%	99.6%
**17**	IL-6	0.2%	99.8%
**18**	Ferritin	0.2%	100.0%

sTNFR: soluble tumor necrosis factor α receptor; MCP-1: Monocyte Chemoattractant Protein-1; hsCRP: High-Sensitivity C-Reactive Protein; TNF-α: Tumor necrosis factor α; IgM: Immunoglobulin M; VCAM-1: Vascular cell adhesion molecule-1; IgA: Immunoglobulin A; IgG: Immunoglobulin G; IL-6: Interleukin-6.

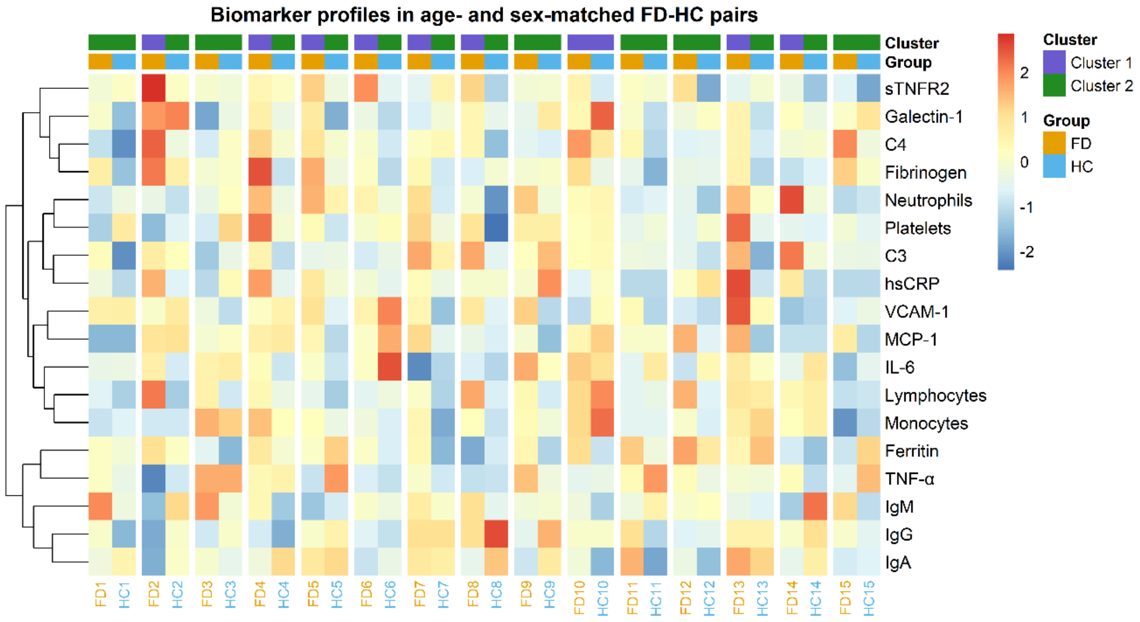

Figure S1. Side-by-side heatmap of standardized biomarker levels in the fifteen age- and sex-matched Fabry disease (FD)–healthy control (HC) pairs. Rows correspond to biomarkers and columns to individual subjects, arranged as matched pairs so that each patient with FD is displayed immediately adjacent to its matched control (separated by vertical gaps); color intensity reflects relative biomarker levels, with red indicating higher and blue indicating lower standardized values. hsCRP: High-Sensitivity C-Reactive Protein; IgA: Immunoglobulin A; IgM: Immunoglobulin M; IgG: Immunoglobulin G; IL-6: Interleukin-6; MCP-1: Monocyte Chemoattractant Protein-1; TNF-α: Tumor necrosis factor α; sTNFR2: soluble tumor necrosis factor α receptor 2; VCAM-1: Vascular cell adhesion molecule-1.

Table S3. Demographic, genetic, and clinical characteristics of patients with Fabry disease according to cluster assignment

**Variable**	**Cluster 1** (n = 9 FD)	**Cluster 2** (n = 6 FD)
**Age (years), median [IQR]**	45 [40–58]	43 [32–44]
**Sex, n (%)**		
Female	6 (67%)	5 (83%)
Male	3 (33%)	1 (17%)
**FD phenotype, n (%)**		
Classical	6 (67%)	3 (50%)
Later-onset	3 (33%)	3 (50%)
**Major organ involvement, n (%)**	6 (67%)	3 (50%)
Renal	6 (67%)	3 (50%)
Cardiac	4 (44%)	1 (17%)

Cerebrovascular	3 (33%)	0 (0%)
**Organ-involvement pattern, n (%)**		
No involvement	3 (33.3)	3 (50%)
Renal only	2 (22%)	2 (33%)
Renal + cardiac	1 (11%)	1 (17%)
Renal + cardiac + cerebrovascular	3 (33%)	0 (0%)
**FD treatment (treated), n (%)**	5 (56%)	2 (33%)

Renal involvement: proteinuria or eGFR < 60 mL/min (CKD-EPI). Cardiac involvement: left ventricular hypertrophy or cardiac arrhythmia. Cerebrovascular involvement: ischemic lesions on MRI, history of stroke or transient ischemic attack.
